# Adherence to CPAP Therapy in Obstructive Sleep Apnea: A Prospective Study on Quality of Life and Determinants of Use

**DOI:** 10.3390/ejihpe14090163

**Published:** 2024-08-27

**Authors:** Karla Milinovic, Ivana Pavlinac Dodig, Linda Lusic Kalcina, Renata Pecotic, Natalija Ivkovic, Maja Valic, Zoran Dogas

**Affiliations:** 1 Department of Family Medicine, Split-Dalmatia Health Center, 21000 Split, Croatia; karla.st.123@gmail.com; 2 Department for Neuroscience, University of Split School of Medicine, 21000 Split, Croatia; linda.lusic@mefst.hr (L.L.K.); renata.pecotic@mefst.hr (R.P.); nivkovic96@gmail.com (N.I.); maja.valic@mefst.hr (M.V.); zdogas@gmail.com (Z.D.)

**Keywords:** obstructive sleep apnea, OSA, severe OSA, CPAP, adherence, quality of life

## Abstract

Obstructive sleep apnea (OSA) often goes unrecognized despite common symptoms, such as excessive daytime sleepiness, fatigue, and impaired quality of life (QoL). Continuous positive airway pressure (CPAP) is the gold standard treatment for OSA, but optimal daily usage and time needed for observable effects remain unclear. This study aimed to investigate the short-term effects of CPAP on daytime sleepiness and QoL in patients with severe OSA. Medical records were collected from 87 patients with severe OSA who initiated CPAP therapy. Also, validated questionnaires were used before and after one month of CPAP to analyze QoL—the Calgary Sleep Apnea Quality of Life Index (SAQLI), the Cues to CPAP Use Questionnaire (CCUQ), and daytime sleepiness—the Epworth Sleepiness Scale (ESS). Multiple regression analysis was conducted to identify predictors of CPAP usage. Of the total participants aged 55.6 ± 12.5, 77% were males, and 62% were CPAP adherent. Reductions in daytime sleepiness (ESS) were noted, as well as improvements in both overall QoL (SAQLI) and specifically in the domains of daily functioning, social interactions, emotional well-being, and symptom perception. Important cues for CPAP usage recognized by patients were physicians’ instructions and physicians’ concern regarding their patients’ condition. Furthermore, multiple regression revealed higher SAQLI scores and lower ESS scores as positive predictors of CPAP usage, along with lower AHI after one month of CPAP being associated with sufficient adherence.

## 1. Introduction

It has been estimated that approximately 4.7% of the worldwide population has obstructive sleep apnea (OSA), with 2% of cases being severe [1,2,3]. Characterized by recurrent collapses of the upper airway during sleep, OSA usually presents with snoring and reduced airflow due to decreased activity of the upper airway dilator muscles and a narrowed airway [4,5,6]. Due to multiple interruptions of sleep by breathing cessations, signs and symptoms of OSA include non-restorative sleep, excessive daytime sleepiness, fatigue, tiredness, lack of energy, abrupt awakenings, and morning headaches [7,8]. Furthermore, OSA occurs significantly more frequently in patients with conditions such as arterial hypertension, hyperlipidemia, type 2 diabetes mellitus, atrial fibrillation, stroke, obesity, posttraumatic stress disorder, and major depressive disorder [9,10,11].

Several studies have confirmed the significant impairment of the quality of life (QoL) in OSA patients [12,13]. Although the concept of QoL is challenging to define, it encompasses overall well-being involving both objective and subjective determinants related to physical, material, social, and emotional aspects [14]. Furthermore, improving QoL might result in superior patient care and rehabilitation, as well as in reduced disease symptoms [15]. Additionally, the concept of health-related QoL is considered to reflect the impact of illness and treatment on patient disability and daily functioning [14,15]. Due to the complex and comprehensive nature of QoL, numerous instruments are employed to assess it, primarily developed based on empirical considerations [15]. The Calgary Sleep Apnea Quality of Life Index (SAQLI) is specifically designed to examine the health-related QoL in OSA patients, and this enables measurement of changes in patients’ conditions following therapeutic interventions [13,16].

In addition to therapeutic recommendations for lifestyle changes and other effective treatments which include oral appliances that cause forward protrusion of the mandible during sleep, surgical modifications of the pharyngeal soft tissue or facial skeleton, and hypoglossal nerve stimulation, continuous positive airway pressure (CPAP) remains the gold standard treatment for moderate to severe OSA patients [8,17]. Furthermore, it has been shown that CPAP reduces daytime sleepiness and improves health-related QoL [18]. However, CPAP adherence in OSA patients often remains unsatisfactory, with some studies revealing that up to 80% of patients use CPAP for less than 4 h per night [8,17,18]. The necessary duration of device usage to achieve optimal therapeutic effects is yet to be determined, and most studies have focused on the therapy’s effectiveness in improving the QoL after several months of usage [17,18] even though symptoms such as daytime sleepiness may reduce as early as following one month of therapy initiation [19].

Therefore, the aim of our study was to assess the effects of one month of CPAP treatment on the QoL (SAQLI) and daytime sleepiness (Epworth sleepiness scale, ESS) in severe OSA patients. Additionally, using the Cues to CPAP Use Questionnaire (CCUQ), we aimed to identify the reasons contributing the most to patients’ initiation of therapy. 

## 2. Materials and Methods

### 2.1. Ethics

The research followed the principles of the Helsinki Declaration [20] and other relevant guidelines, providing ethical conduct and the participants’ safety. Before study enrollment, all participants signed the informed consent, and confidentiality was maintained throughout the study. The Ethics Committee of the University of Split School of Medicine approved the study (protocol code: 003-08/22-03/0003).

### 2.2. Patients

A total of 87 consecutive severe OSA patients ranging from 27 to 80 years of age, diagnosed between 1 October 2022 and 31 May 2023 at the Sleep Medicine Center of the University of Split School of Medicine and the University Hospital of Split, were included in the study. Patients were assessed with either whole-night polysomnography (PSG) or polygraphy (PG), and severe OSA was defined as an apnea–hypopnea index (AHI) of ≥30 [8]. All patients underwent an examination by an otolaryngologist before PSG or PG testing at our reference center. The specialist assessed the upper airway, as well as the characteristics of the nose, mouth, and throat, and determined whether patients were suitable as candidates for CPAP therapy. The patients who were not suitable for CPAP therapy due to unfavorable characteristics of the upper airways and had undergone surgery prior to CPAP therapy were not included in the study. Additionally, the inclusion criteria were an age of 18 years or older and the necessary physical and mental capability to adhere to the research protocol, which was assessed by the attending physician.

### 2.3. Questionnaires

A total of three validated questionnaires were used: the Epworth Sleepiness Scale (ESS) [21,22,23], the Calgary Sleep Apnea Quality of Life Index (SAQLI) [16,24], and the Cues to CPAP Use Questionnaire (CCUQ) [25].

The ESS assesses participants’ possibility of falling asleep in eight common daily situations and is widely used in multiple languages and clinical settings [21,22]. The participants respond on a scale from 0 (never doze) to 3 (high chance of dozing), with a total score ranging from 0 to 24 [23], and a higher score indicating increased daytime sleepiness [23].

The SAQLI evaluates the health-related quality of life in patients with OSA, primarily for research purposes rather than routine clinical practice [16,24]. It consists of 45 questions distributed across distinct domains: daily functioning (A), social interactions (B), emotional functioning (C), patient-selected or nominated symptoms potentially due to OSA (D), and treatment-related symptoms (E). Responses for Domain E are exclusively filled out after therapy implementation. Each question is rated on a scale from 1 (indicating maximum impairment) to 7 (indicating no impairment) [16,24]. The average score is calculated for each specific domain. To determine the overall SAQLI score, the result of Domain E is subtracted from the score of each domain, and the average is subsequently computed. This unique characteristic of the questionnaire incorporates therapy side effects [16,24].

The final questionnaire, CCUQ, aims to identify the primary factors influencing a patient’s decision to commence CPAP therapy. The questionnaire consists of nine items or potential reasons, to which respondents provide answers on a scale from 0 (not at all) to 3 (extremely important) [25]. 

### 2.4. Data Collection and Statistical Analysis

Before undergoing PSG or PG procedures, demographic data, medical history, and comorbidity information were collected, alongside the completion of the ESS. Following this, all patients underwent either the whole-night PSG (Alice 6, Philips Respironics, Eindhoven, The Netherlands) or PG recording (Alice NightOne, Philips Respironics, Eindhoven, The Netherlands and SOMNOcheck2, Weinmann, Hamburg, Germany). Obtained data were manually scored and OSA was diagnosed according to AASM diagnostic criteria and ESRS guidelines [26].

**Figure 1:**
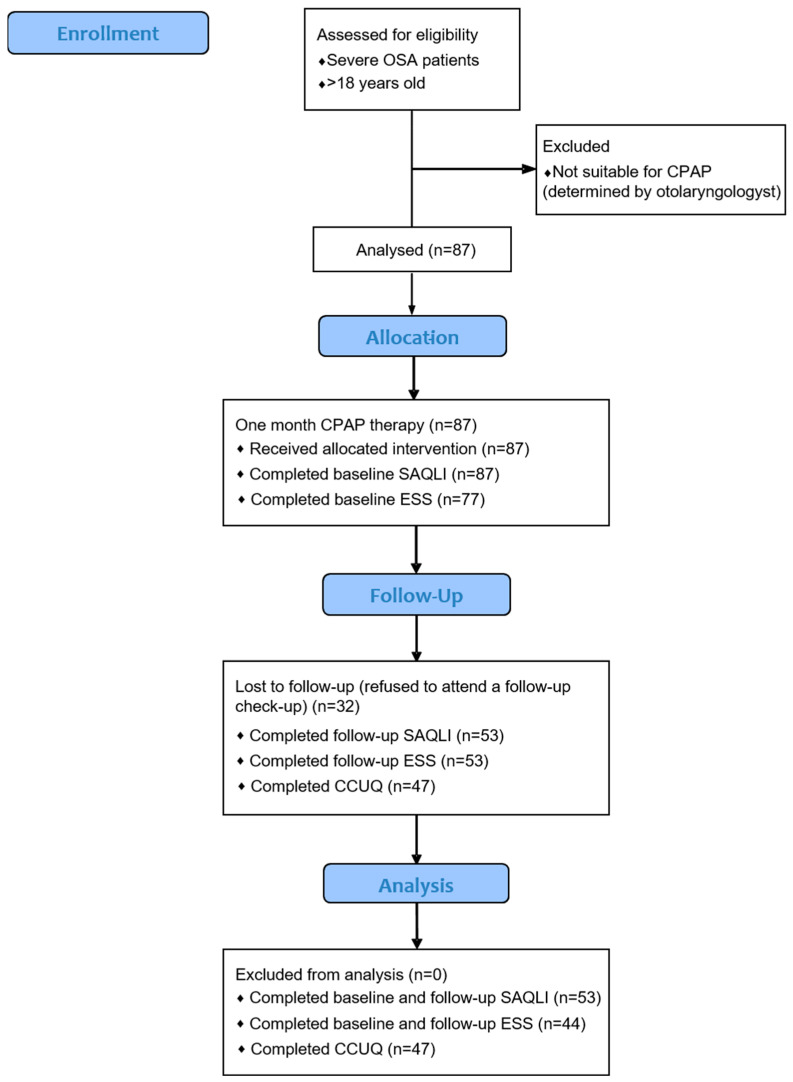
A flowchart diagram of the study. (OSA: obstructive sleep apnea, CPAP: continuous positive airway pressure, ESS: Epworth Sleepiness Scale, SAQLI: Calgary Sleep Apnea Quality of Life Index, CCUQ: Cues to CPAP Use Questionnaire).

Before initiating CPAP therapy, all 87 patients were asked to complete the pen-and-paper SAQLI questionnaire. A total of 55 patients (63.2%) agreed to attend a follow-up check-up after one month of CPAP usage, during which data from the CPAP memory cards showing usage were extracted. Participants also completed the CCUQ as well as the follow-ups of ESS and SAQLI questionnaires. Follow-up data were collected from 47 participants for CCUQ, 53 participants for ESS, and 53 participants for the SAQLI (Figure 1). 

Data analysis was performed in Microsoft Excel, version 13.0 (Microsoft Corporation, Redmond, WA, USA), and SPSS Statistics 23.0 (IBM Corporation, Armonk, NY, USA). Continuous data were presented as mean ± standard deviation (SD), while categorical variables were expressed as absolute numbers and percentages. Data distribution normality was tested by the Shapiro–Wilk test. For the comparison of continuous variables, the *t*-test for independent samples was used, while the χ^2^-test was used for nominal scale data. To assess the differences in questionnaire results before and after CPAP therapy, repeated measures ANOVA was conducted. The ANOVA analysis on SAQLI was conducted for 53 patients who completed questionnaires before and after CPAP. However, considering that the initial ESS was filled out by 77 patients, the ANOVA analysis was conducted on the 44 who completed the ESS both before and after one month of CPAP usage (Figure 1). Patients were categorized based on CPAP compliance (percentage of days with CPAP usage ≥4 h per day). According to the American Academy of Sleep Medicine (AASM) recommendations, which are consistent with the existing literature, those who used CPAP ≥4 h per day on more than 70% of days over one month were classified as adherent, whereas all other participants were considered non-adherent [27,28]. The odds ratio was calculated for the association between CCUQ and CPAP compliance, with significance assessed using the Fisher exact test. The association of CPAP compliance with the following variables: AHI, ESS score before CPAP initiation, and total SAQLI score before CPAP usage were analyzed by multiple regression. We also analyzed several other regression models that included other available parameters. However, we have chosen to present the current model, as it was the one that reached statistical significance. A *p*-value of <0.05 was considered statistically significant.

Reliability analysis for the daily functioning domain, the social interactions, and the emotional functioning domain of SAQLI, including the item-total statistics for each of the items included in the domain is reported in Supplementary Tables S1–S3. The same reliability analysis is reported for all of the items in the Cues to CPAP Use Questionnaire (CCUQ) in Supplementary Table S4. Tables including Cronbach’s alpha value of the scales, Cronbach’s alpha based on standardized items, as well as the total numbers of items included are also added to the reliability analysis (Supplementary Tables S1–S4).

## 3. Results

A total of 87 patients diagnosed with severe OSA were enrolled in this study. The mean age of the study participants was 55.6 ± 12.5 years. The majority were male (77%), and the body mass index was 33.2 ± 7.4 kg/m^2^ (Table 1). Polysomnography or polygraphy results revealed an AHI of 50.5 ± 19.1 with a mean oxygen saturation of 92.8% ± 3.6%.

After one month of CPAP usage, 55 patients consented to a regular follow-up examination, when data from CPAP memory cards were extracted (Table 2 and Supplementary Figure S1). Based on the percentage of days with CPAP usage ≥ 4 h per day, 21 of them, or 38.2%, were classified as non-compliant, while 61.8% were compliant. In accordance with that, CPAP-non-compliant patients had shorter average usage than compliant patients in hours on both all days (3.3 ± 1.2 vs. 6.3 ± 1.2 h, *p*-value < 0.001) and on days when CPAP was used (4.3 ± 1.2 vs. 6.6 ± 1.2 h, *p*-value < 0.001), as assessed by *t*-test for independent samples. Both non-compliant and compliant patients averagely used CPAP on more than 70% of days. However, the compliant group used it on 94.4% ± 9% of days, while non-compliant OSA patients used it on 78.3% ± 23.1% of days (*p*-value < 0.001, *t*-test for independent samples). The mean AHI during CPAP usage was found to be 4.7 ± 4.6. AHI among non-compliant patients was significantly higher than among compliant patients (6.5 ± 6.3 vs. 3.5 ± 2.8, respectively; *p*-value = 0.018, calculated by independent samples *t*-test).

At the check-up after one month of CPAP therapy, the scores for all domains of SAQLI were significantly lower, including daily functioning (domain A), social interactions (domain B), emotional functioning (domain C), and patient-selected or nominated symptoms potentially due to OSA (domain D), indicating better quality of life (Table 3 and Supplementary Figure S2). Additionally, the total score after one month of CPAP therapy was significantly lower than the pre-CPAP score (3.4 ± 1.1 vs. 1.7 ± 0.9, respectively, *p*-value < 0.001 assessed by repeated measures ANOVA). Patients exhibited significantly lower check-up ESS scores, indicating lower daytime sleepiness (7.2 ± 5.1 vs. 4.6 ± 4.2, *p*-value = 0.004, repeated measures ANOVA).

**Figure 2:**
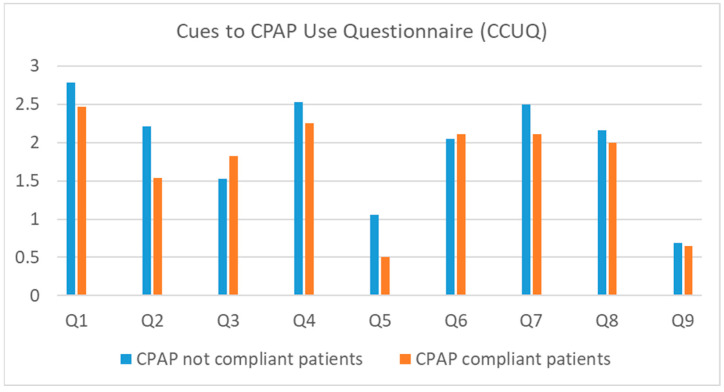
The frequency of endorsement for each item on the Cues to CPAP Use Questionnaire (CCUQ) concerning CPAP compliance (n = 47). (CPAP compliant: CPAP used ≥4 h per day on more than 70% of days, CPAP not compliant: CPAP was not used ≥4 h per day on more than 70% of days, Q: question).

As shown in Figure 2, non-compliant patients exhibited higher endorsement rates on all items of the CCUQ except for Q3 (“I started using CPAP because my partner could not sleep because of my snoring”) and Q6 (“My partner encouraged me to start using CPAP”).

Responses to the CCUQ were grouped into two categories: “not at all” and “little important” vs. “moderately important” and “extremely important”. The association between CPAP compliance and the relevance of the specific cue is shown in Table 4. It was demonstrated that a statistically significant difference was only observed for Q2 (“I started using CPAP because I was worried about my heart”), where answers “moderately or extremely important” were associated with subsequent CPAP non-compliance (OR: 0.263 (95% CI: 0.028–2.443); *p*-value = 0.022). Therefore, it appears that patients starting to use CPAP due to heart concerns have a low probability of being in the compliant group. Overall, patients most frequently reported the relevance of Q1, indicating the relevance of physicians’ instructions, and Q4, indicating the physicians’ worry regarding patients’ condition.

Multiple regression analysis confirmed both ESS and SAQLI before CPAP in patients diagnosed with severe OSA were significantly associated with CPAP compliance, explaining 13.7% of the variability, while AHI was not (Table 5). In more detail, lower levels of daytime sleepiness and patients’ subjective perception of worse QoL measured before CPAP initiation were significant predictors of better CPAP adherence. However, OSA severity assessed with AHI before CPAP therapy was not a significant predictor of CPAP compliance.

## 4. Discussion

The results of this study showed that severe OSA patients had an improvement in OSA-related quality of life and reduced daytime sleepiness following one month of CPAP therapy. This improvement was established in daily functioning, social interactions, emotional well-being, and symptom perception, which emphasizes the benefits of effective OSA treatment on patients’ quality of life.

It has been shown that CPAP is efficient in mitigating respiratory events during sleep in severe OSA patients, and it also has favorable effects on alleviating symptomatic manifestations of OSA and improving daytime functioning [7,12,13]. This was supported by the results of the present study showing a significant reduction in AHI and daytime sleepiness among compliant patients.

Previous studies reported lower QoL with poor sleep quality, excessive daytime sleepiness, dizziness, headaches, decreased concentration, mood changes, impaired hearing, and voice alteration among OSA patients when compared to the general population [7,12,17,29,30,31,32]. In OSA patients, QoL is closely associated with factors such as age, stress, lifestyle, depression, BMI, and overall health status [30]. Numerous studies have investigated the impact of various factors on the broad concept of QoL, yet the distinctive influence of individual factors remains unclear [30]. In recent years, improvement in QoL has become a relevant aim in OSA treatment [17]. CPAP successfully alleviates symptoms and reduces disease severity [17,30,32,33,34], and improves QoL due to improvement in neurocognitive function, sleep quality and restfulness, emotional well-being, symptoms of depression, and social functioning [30,31,32]. This is in accordance with the results of the current study, showing an improvement across all analyzed domains of QoL, including daily functioning, social interactions, emotional functioning, and patient-selected symptoms.

In the present study, where the QoL was assessed with an OSA-specific SAQLI questionnaire, the overall score significantly decreased within only one month, surpassing the clinically meaningful score change. Improvements were observed in daily functioning, social interactions, emotional well-being, and symptom perception. Differences between SAQLI and other QoL questionnaires include its OSA-specificity, and considering treatment-related symptoms [7,13,16]. Furthermore, SAQLI has a high correlation with the widely used, disease-nonspecific 36-Item Short Form Health Survey (SF-36) [13]. A recent meta-analysis found that SAQLI scores remained improved at both 3 and 12 months with CPAP therapy [7], while previous studies also report improvements at 6 months, but not at 2 months of CPAP therapy [13]. Still, the observed 1.7-point improvement in our study has been recognized within one month of CPAP therapy, and such findings may be attributed to the fact that all patients had severe OSA with an average AHI of 50. 

This study included severe OSA patients, predominantly middle-aged men with increased BMI, reflecting previously established OSA risk factors [35]. Based on CPAP usage data, 38.2% of enrolled severe OSA patients were non-compliant, reflecting the world trends [18,36] and highlighting the challenges in achieving optimal CPAP adherence [5,18]. OSA is considered a disease with one of the poorest adherence rates to therapy, with non-adherence rates ranging from 29% to 83% [18]. Consequently, recent research has focused on strategies to increase CPAP adherence, including educational materials, regular check-ups, motivational therapy, telephone calls, online consultations, or home visits [5,18,37,38]. The effectiveness of these interventions, however, is questionable [5,18]. Behavioral therapy, such as motivational interviewing, has been proven to be one of the most effective for CPAP compliance [18]. It requires highly educated staff and considerable one-on-one time with patients [18]. Educational materials, whether in written or video format, although incomprehensible to some patients, offer a relatively straightforward approach and, according to some authors, significantly increase adherence [18]. While our patients were not candidates for surgical treatment, some studies suggest that it can improve CPAP adherence in patients for whom it is indicated. Nasal surgery in patients with nasal obstruction, according to some authors, reduces therapeutic CPAP device pressure and increases the use of CPAP in certain patients [39]. Moreover, adherence after just one month serves as a good predictor of future adherence [40], providing an opportunity for targeted interventions in those exhibiting poor adherence in the short term. Nonetheless, additional research is imperative to ascertain the most effective approaches. It is favorable that in our study, even patients classified as non-compliant still attempted to use CPAP, and they might become compliant users in the coming months. One might presume that just one month of usage was not sufficient for them to adapt to the device, hence they had poorer compliance results.

To provide a comprehensive analysis of CPAP compliance, the current study also assessed the patient attitudes and motivations influencing CPAP compliance using the CCUQ questionnaire. A significant predictor of non-compliance in our study was patients’ concern regarding cardiovascular health. Previous research has presented conflicting views regarding hypertension as an indicator of cardiovascular health concerning CPAP compliance [41]. While some authors consider it a predictor of compliance, others suggest it indicates non-compliance, with certain studies showing no correlation at all [41]. Various other predictors of CPAP compliance have also demonstrated variability depending on the setting and study group [3,41]. Nevertheless, established predictors associated with higher adherence include older age, female sex, ESS > 10, severe OSA (AHI > 30), health insurance coverage, lower rates of depression and insomnia, lower SpO2 levels, and the use of hypnotics [3,41]. A recent systematic review emphasizes patient personality and their beliefs and cognitions as significant predictors of adherence [42], as well as the value patients place on their health and their self-efficacy, referring to their confidence and ability to self-manage CPAP treatment [42,43].

Our multiple regression analysis revealed lower ESS scores and higher SAQLI domains measured before CPAP initiation as significant predictors of CPAP compliance. Namely, patients’ subjective perception of worse QoL was a significant predictor of better adherence. Our finding regarding ESS contrasts with the existing literature [19,44], suggesting that in severe OSA patients, domains of QoL would have a stronger impact on better CPAP adherence, rather than daytime sleepiness measured by ESS. Furthermore, AHI levels before CPAP therapy were not significant predictors of CPAP compliance. While it is well-established that CPAP significantly reduces AHI [19], the fact that adherence was not associated with AHI in our patients emphasizes the necessity of considering additional factors that might influence compliance in this population. These factors might be associated with patient-specific characteristics, such as belonging to a certain OSA phenotype cluster [45], which significantly influences the variability in treatment adherence and thus merits further investigation.

Limitations of the study are a relatively small sample size, a single-center setting, and a short follow-up period of only one month, which could limit the generalization of findings and impact the results. Extended follow-up periods might provide comprehensive data, possibly yielding practical guidelines for improvement of the long-term CPAP compliance. Thus, future research should include larger, multicenter studies and focus on additional determinants of CPAP compliance, such as psychosocial factors, socioeconomic status, and healthcare system-related barriers. Moreover, longitudinal studies with extended follow-up periods could evaluate long-term treatment effects on QoL and adherence patterns among patients undergoing CPAP therapy. Finally, it is important to note that patients did not undergo drug-induced sleep endoscopy (DISE), due to the complexity of the procedure in terms of required personnel and time. It is otherwise a highly valuable diagnostic tool for patients with sleep-disordered breathing, as it evaluates the sites of upper airway obstruction and vibrations. Since all participants in our study were candidates for CPAP therapy, performing this procedure was deemed unnecessary. Nevertheless, it should be noted that this diagnostic method could be highly beneficial for patients who do not tolerate CPAP therapy or are not suitable candidates for it [8].

## 5. Conclusions

In conclusion, this study provides valuable insights into the quality of life, compliance patterns, and reasons relevant for good CPAP adherence among severe OSA patients undergoing CPAP therapy. Furthermore, the study highlights the importance of addressing patient-centered factors in promoting CPAP adherence and the use of disease-specific questionnaires such as SAQLI. Tailored interventions aimed at optimizing CPAP adherence and enhancing treatment outcomes among patients with severe OSA should be used to improve long-term treatment compliance and efficacy.

## Figures and Tables

**Table 1:** Demographic, anthropometric characteristics, smoking status, and comorbidities of severe obstructive sleep apnea (OSA) patients with polysomnography (PSG) or polygraphy (PG) results.

Parameters	Total Patients (n = 87)
Age (years, mean, and SD)	55.6 ± 12.5
Sex	
Men (n, %)	67 (77%)
Women (n, %)	20 (23%)
Height (cm, mean, and SD)	179.3 ± 9.2
Weight (kg, mean, and SD)	108.9 ± 24.7
Body mass index (kg/m^2^, mean, and SD) *	33.2 ± 7.4
18.5 ≤ 25 (n, %)	5 (6.5%)
25 ≤ 30 (n, %)	19 (24.7%)
30 ≤ 35 (n, %)	24 (31.2%)
35 ≤ 40 (n, %)	20 (26%)
≥40 (n, %)	9 (11.7%)
Neck circumference (cm, mean, and SD)	45.4 ± 5.0
Waist circumference (cm, mean, and SD)	118.0 ± 16.0
Hip circumference (cm, mean, and SD)	115.6 ± 12.4
Apnea–hypopnea index (/hour, mean, and SD)	50.5 ± 19.1
Oxygen desaturation index (/hour, mean, and SD)	51.0 ± 22.8
Mean saturation (%, mean, and SD)	92.8 ± 3.6
Time below 90% saturation (hours, mean, and SD)	1.4 ± 1.8
Lowest saturation (%, mean, and SD)	72.3 ± 11.8
Smoker (n, %)	25 (28.7%)
Arterial hypertension (n, %)	57 (65.5%)
Diabetes mellitus type II (n, %)	22 (25.3%)
Depression (n, %)	4 (4.6%)
Asthma (n, %)	8 (9.2%)
Gastroesophageal reflux disease (n, %)	17 (19.5%)
Allergic rhinitis (n, %)	10 (11.5%)
Previous rhinoplasty (n, %)	9 (10.3%)
ECOG Performance Status Scale	
0 (n, %)	33 (37.9%)
1 (n, %)	54 (62.1%)
2 (n, %)	0 (0%)
3 (n, %)	0 (0%)
4 (n, %)	0 (0%)
5 (n, %)	0 (0%)

ECOG: Eastern Cooperative Oncology Group, SD: standard deviation. * n = 77 available data for BMI, none of the participants had BMI ≤ 18.5.

**Table 2:** The data from the CPAP memory cards after one month of usage in compliant and non-compliant patients.

Parameters	Total (n = 55)	CPAP Non-Compliant (n = 21)	CPAP Compliant (n = 34)	*p*-Value ^†^
Average usage on all days (hours) *	5.1 ± 1.9	3.3 ± 1.2	6.3 ± 1.2	<0.001
Average usage on days when CPAP was used (hours) *	5.7 ± 1.7	4.3 ± 1.2	6.6 ± 1.2	<0.001
Total days with device usage (n) *	26.8 ± 7.4	23.6 ± 10.3	28.8 ± 3.5	0.011
Percentage of days with device usage (%)	88% ± 17.8%	78.3% ± 23.1%	94.4% ± 9%	<0.001
Total days with CPAP usage ≥ 4 h (n)	20.5 ± 9.3	10.7 ± 5.8	26.5 ± 4.8	<0.001
Percentage of days with CPAP usage ≥ 4 h (%)	69.8% ± 28.7%	37.9% ± 19%	89.4% ± 9.5%	<0.001
Average apnea–hypopnea index (n)	4.7 ± 4.6	6.5 ± 6.3	3.5 ± 2.8	0.018

All data are shown as mean ± standard deviation. CPAP: continuous positive airway pressure, CPAP compliant: CPAP used ≥4 h per day on more than 70% of days, CPAP non-compliant: CPAP was not used ≥4 h per day on more than 70% of days. * n = 53 available data, ^†^ independent samples *t*-test (*p*-value < 0.05 was considered statistically significant).

**Table 3:** Calgary Sleep Apnea Quality of Life Index (SAQLI) and Epworth Sleepiness Scale (ESS) results before and after one month of CPAP usage (n = 53).

Parameters	Before CPAP	After One Month of CPAP	*p*-Value *
Domain A	2.9 ± 1.2	2.1 ± 1.2	<0.001
Domain B	2.9 ± 1.2	2.1 ± 1.2	<0.001
Domain C	2.8 ± 1.3	2.2 ± 1.1	<0.001
Domain D ^†^	5.2 ± 1.7	3.3 ± 1.7	<0.001
Domain E ^†^	-	2.8 ± 1.5	-
Total SAQLI ^†^	3.4 ± 1.1	1.7 ± 0.9	<0.001
Domain F I ^†^	-	7.0 ± 2.4	-
Domain F II ^†^	-	3.6 ± 3.3	-
Epworth Sleepiness Scale ^‡^	7.2 ± 5.1	4.6 ± 4.2	0.004

All data are shown as mean ± standard deviation. CPAP: continuous positive airway pressure. * Repeated measures ANOVA (*p*-value < 0.05 was considered statistically significant), ^†^ n = 50 available data, ^‡^ n = 44 available data.

**Table 4:** The frequency of moderately or extremely important answers for each item on the Cues to CPAP Use Questionnaire (CCUQ) in relation to CPAP compliance; n (%).

Parameters	Total (n = 50)	CPAP Non-Compliant (n = 20)	CPAP Compliant (n = 30)	*p*-Value *	OR (95%CI)	*p*-Value ^†^
Q1 My sleep physician said that I should	44 (88%)	19 (95%)	23 (76.7%)	0.214	0.263 (0.028–2.443)	0.381
Q2 I was worried about my heart	30 (60%)	16 (80%)	14 (46.7%)	0.018	0.219 (0.059–0.810)	0.022
Q3 Partner could not sleep because of my snoring	29 (58%)	10 (50%)	19 (63.3%)	0.349	1.727 (0.548–5.448)	0.393
Q4 My sleep physician was worried about my OSA	42 (84%)	18 (90%)	24 (80%)	0.345	0.444 (0.080–2.465)	0.450
Q5 Advice from a friend/acquaintance (who does not have OSA)	11 (22%)	7 (35%)	4 (13.3%)	0.070	0.286 (0.071–1.155)	0.090
Q6 Partner encouraged me to start CPAP ^‡^	34 (69.4%)	14 (70%)	20 (69%)	0.938	0.952 (0.276–3.286)	1.000
Q7 I was worried about the health consequences of my sleep problem	39 (78%)	17 (85%)	22 (73.3%)	0.329	0.485 (0.112–2.111)	0.489
Q8 I was so tired all of the time	36 (72%)	16 (80%)	20 (66.7%)	0.304	0.500 (0.132–1.896)	0.353
Q9 I was worried that I would have a car accident	10 (20%)	5 (25%)	5 (16.7%)	0.470	0.600 (0.149–0.421)	0.494

All data are shown as n (%). CPAP: continuous positive airway pressure, CPAP compliant: CPAP used ≥4 h per day on more than 70% of days, CPAP non-compliant: CPAP was not used ≥4 h per day on more than 70% of days, Q: question. * Chi square test (*p*-value < 0.05 was considered statistically significant), ^†^ Fisher’s exact test (*p*-value < 0.05 was considered statistically significant), ^‡^ n = 49 available data.

**Table 5:** Multiple regression analysis for predictors of CPAP compliance.

Variable	Unstandardized Coefficients	Standard Error	Standardized Coefficients	T	*p*-Value
Apnea–hypopnea index *	−0.342	0.990	−0.051	−0.345	0.732
Epworth sleepiness scale *	−8.653	3.684	−0.387	−2.349	0.024
Total SAQLI score *	48.263	16.647	0.481	2.899	0.006

CPAP: continuous positive airway pressure, SAQLI: Calgary Sleep Apnea Quality of Life Index. R^2^ = 0.253, adjusted R^2^ = 0.198, F = 4.627, *p*-value = 0.007, *p*-value < 0.05 was considered statistically significant). * Before CPAP.

## Data Availability

The data are contained within the article and the recordings and raw datasets supporting the conclusions of this study will be made available by the corresponding author on request.

## Supplementary Materials

The following supporting information can be downloaded at: https://www.mdpi.com/article/10.3390/ejihpe14090163/s1, Figure S1: The data from the continuous positive airway pressure (CPAP) memory cards after one month of usage in compliant and non-compliant patients; Figure S2: Four (A—daily functioning, B—social interactions, C—emotional functioning, and D—symptoms potentially due to obstructive sleep apnea) domains and the total score of the Calgary Sleep Apnea Quality of Life Index (SAQLI), and Epworth Sleepiness Scale (ESS) results before and after one month of continuous positive airway pressure (CPAP) therapy usage (n = 53); Table S1: Reliability analysis and the Cronbach’s alpha for the daily functioning domain of Calgary Sleep Apnea Quality of Life Index; Table S2: Reliability analysis and the Cronbach’s alpha for the social interactions domain of Calgary Sleep Apnea Quality of Life Index; Table S3: Reliability analysis and the Cronbach’s alpha for the emotional functioning domain of Calgary Sleep Apnea Quality of Life Index; Table S4: Reliability analysis and the Cronbach’s alpha for the Cues to CPAP Use Questionnaire (CCUQ).
