# Supplementary material for: Adherence to CPAP Therapy in Obstructive Sleep Apnea: A Prospective Study on Quality of Life and Determinants of Use
Source: Eur J Investig Health Psychol Educ. 2024 Aug 27;14(9):2463–75. doi: 10.3390/ejihpe14090163 (PMC11431498; doi:10.3390/ejihpe14090163)
Supplement: Supplementary file 1 [file ejihpe-14-00163-s001.zip › Supplementary Figure 1.pdf]

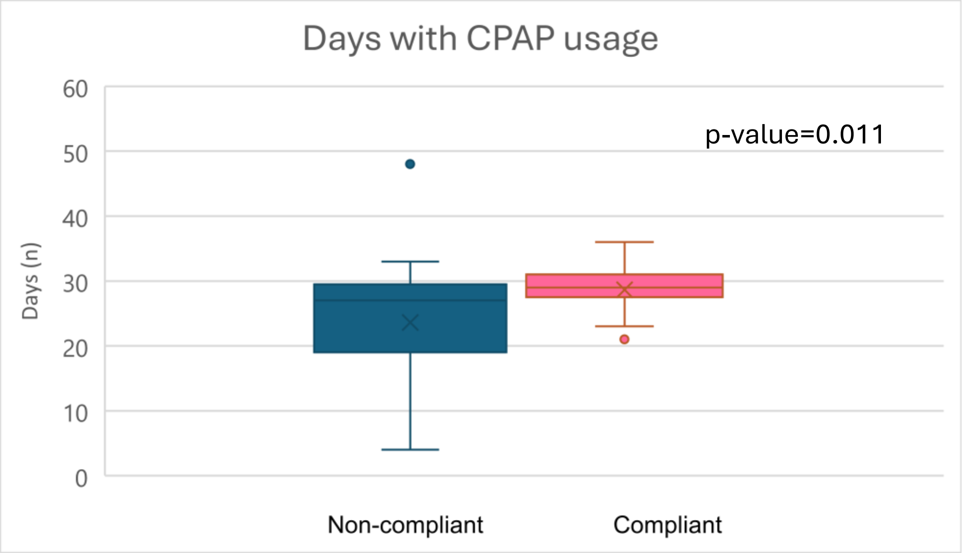
Days with CPAP usage
60
50
40
30
20
10
0
Days (n)
p-value=0.011
Non-compliant
Compliant

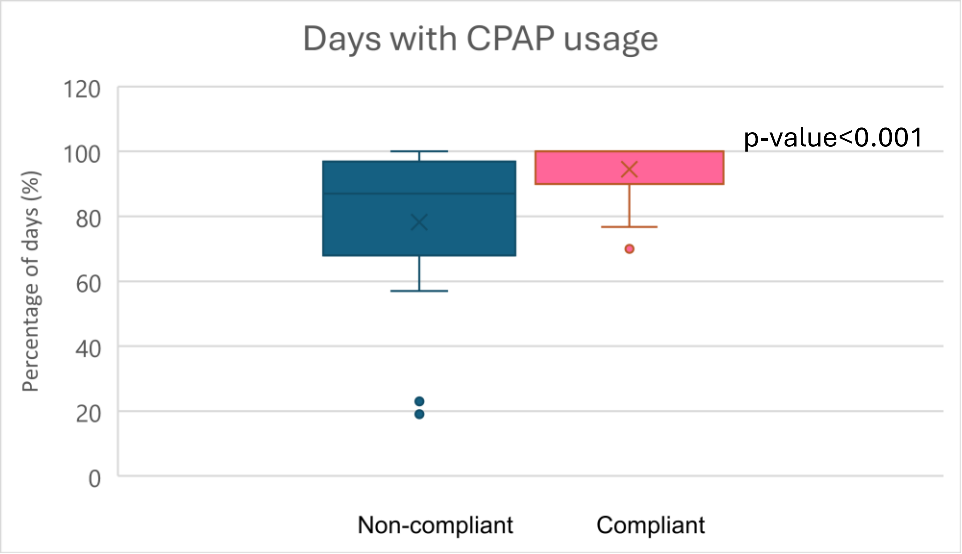
Days with CPAP usage
120
100
80
60
40
20
0
Percentage of days (%)
p-value<0.001
Non-compliant
Compliant

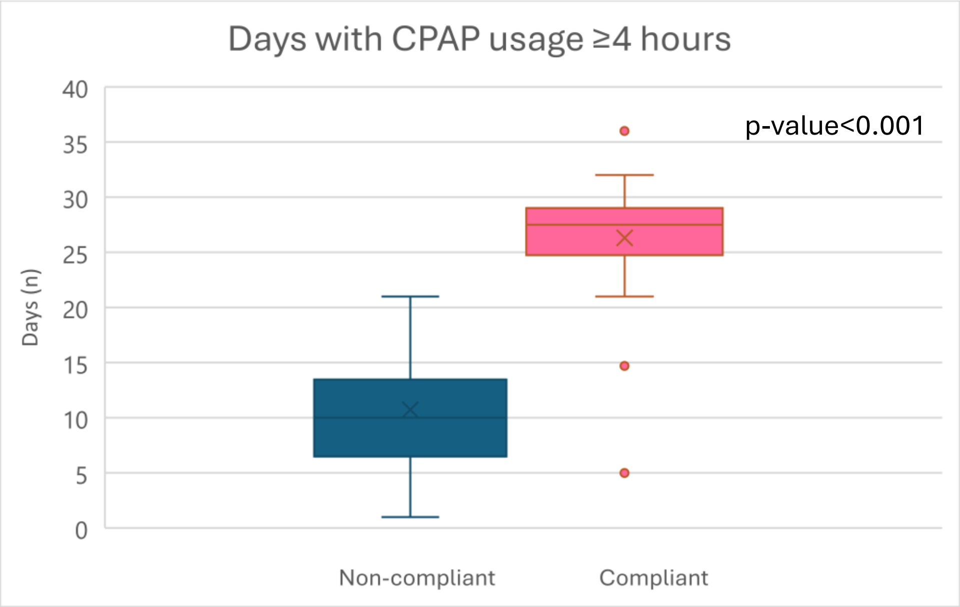
Days with CPAP usage ≥4 hours
40
35
30
25
20
15
10
5
0
Days (n)
p-value<0.001
Non-compliant
Compliant

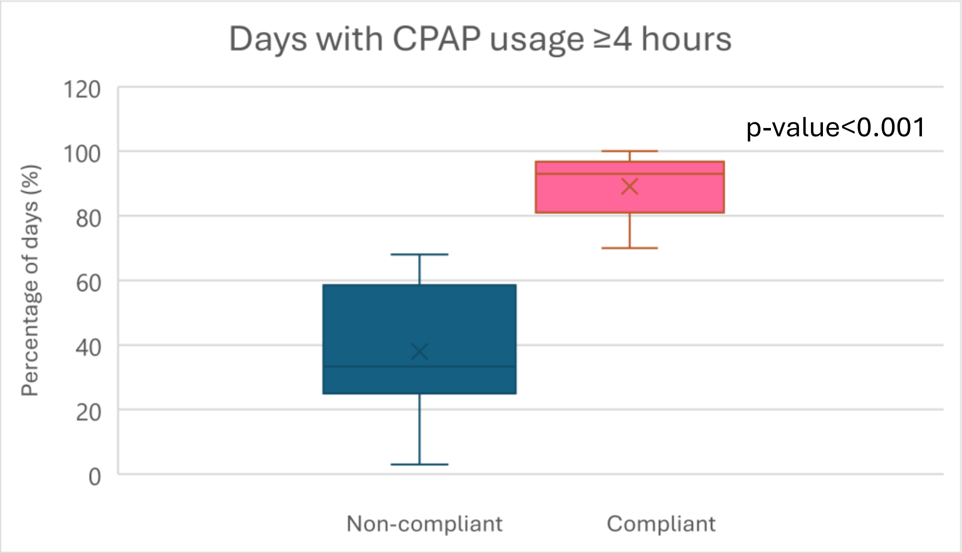
Days with CPAP usage ≥4 hours
Percentage of days (%)
120
100
80
60
40
20
0
p-value<0.001
Non-compliant
Compliant

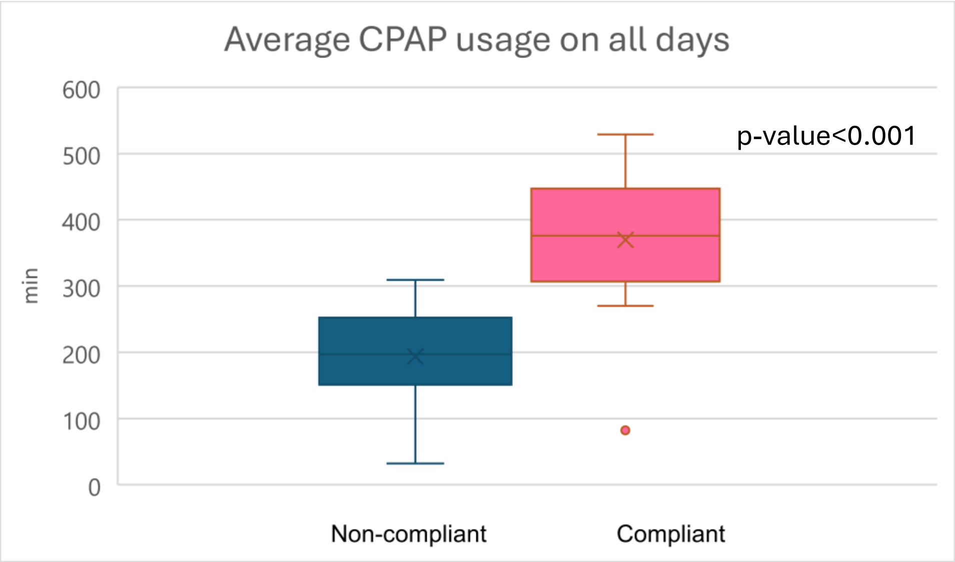
Average CPAP usage on all days
min
600
500
400
300
200
100
0
p-value<0.001
Non-compliant
Compliant

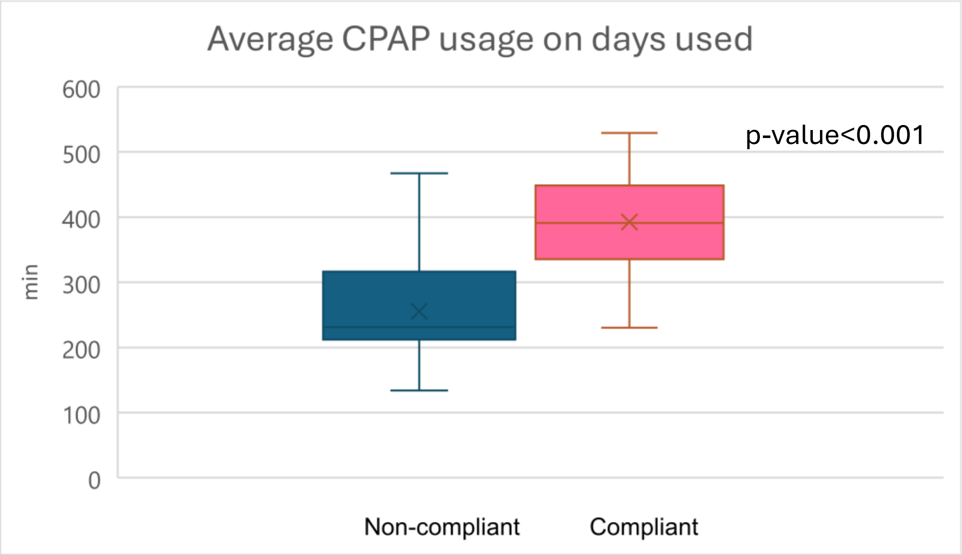
Average CPAP usage on days used
min
600
500
400
300
200
100
0
p-value<0.001
Non-compliant
Compliant

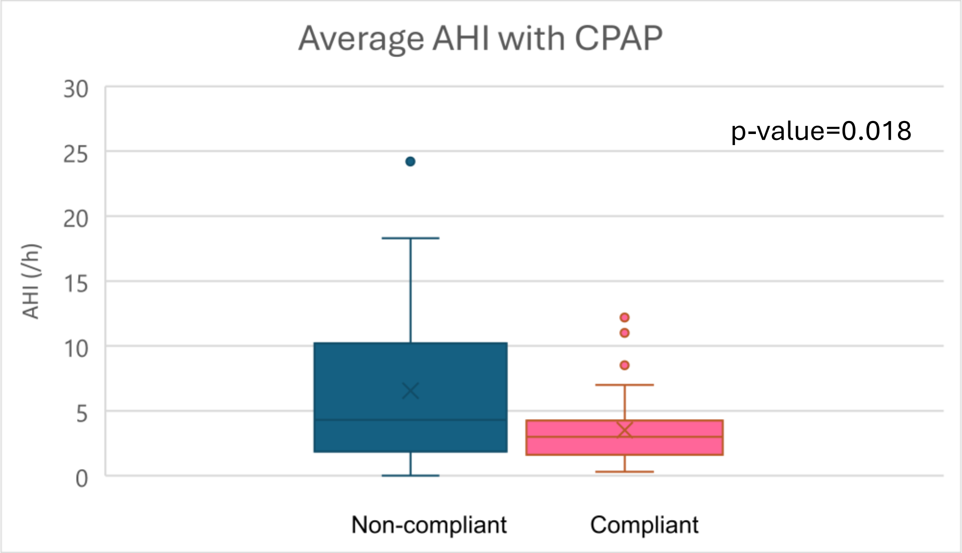

Supplementary Figure 1. The data from the continuous positive airway pressure (CPAP) memory cards after one month of usage in compliant and non-compliant patients.

CPAP: Continuous positive airway pressure, non-compliant: CPAP was not used ≥4 hours per day on more than 70% of days, compliant: CPAP used ≥4 hours per day on more than 70% of days, AHI: apnea-hypopnea index.
