# Supplementary material for: Adherence to CPAP Therapy in Obstructive Sleep Apnea: A Prospective Study on Quality of Life and Determinants of Use
Source: Eur J Investig Health Psychol Educ. 2024 Aug 27;14(9):2463–75. doi: 10.3390/ejihpe14090163 (PMC11431498; doi:10.3390/ejihpe14090163)
Supplement: Supplementary file 1 [file ejihpe-14-00163-s001.zip › Supplementary Figure 2.pdf]

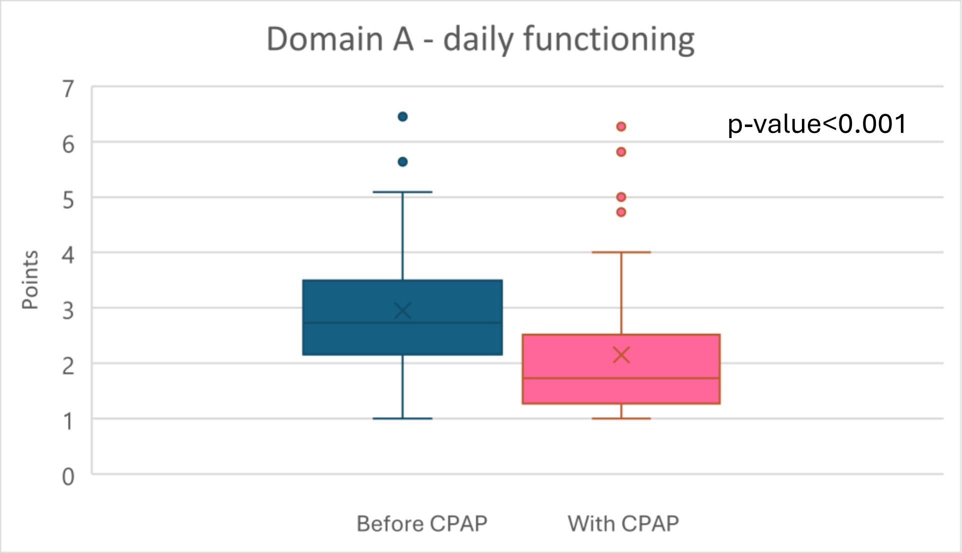

Domain A - daily functioning
7
6
5
4
3
2
1
0
Points
p-value<0.001
Before CPAP
With CPAP

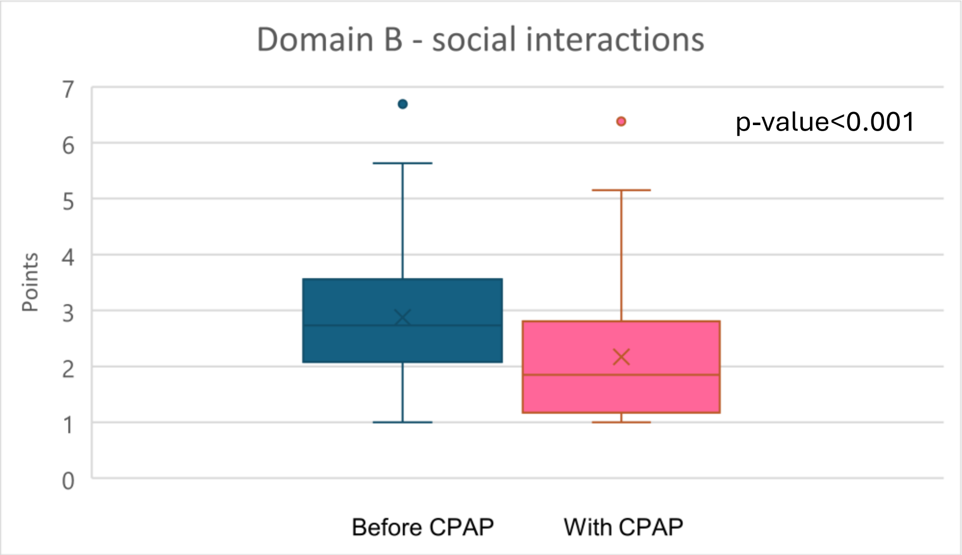

Domain B - social interactions
7
6
5
4
3
2
1
0
Points
p-value<0.001
Before CPAP
With CPAP

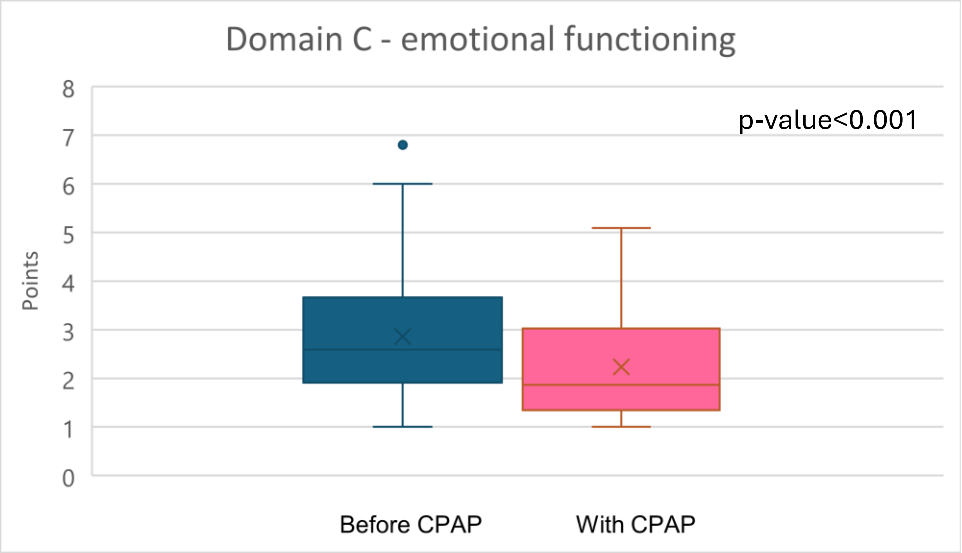

Domain C - emotional functioning
8
7
6
5
4
3
2
1
0
Points
p-value<0.001
Before CPAP
With CPAP

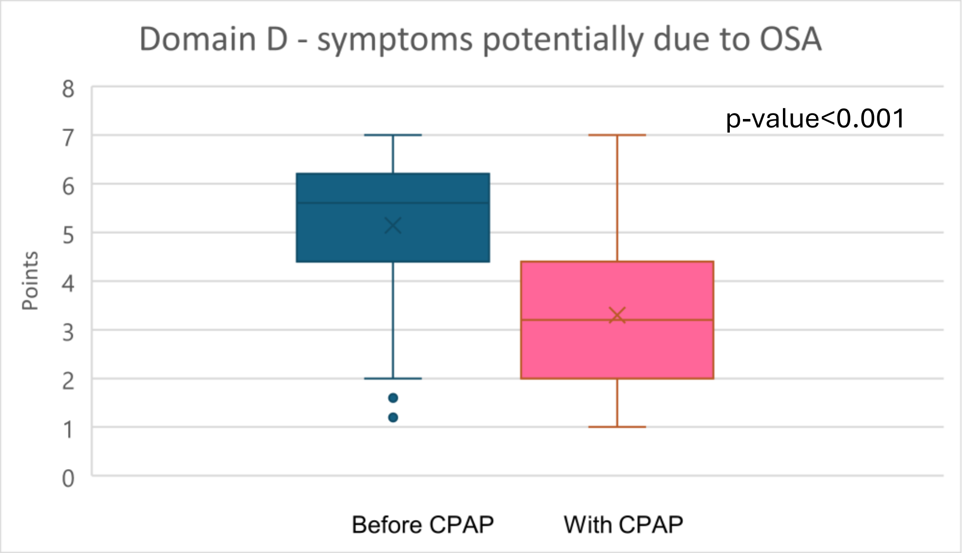
Domain D - symptoms potentially due to OSA
Points
p-value<0.001
Before CPAP
With CPAP

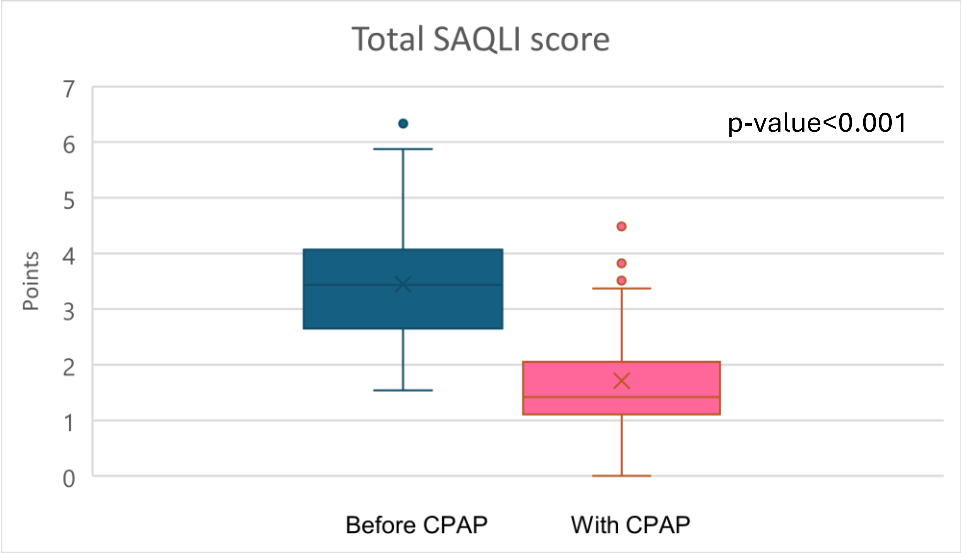
Total SAQLI score
Points
p-value<0.001
Before CPAP
With CPAP

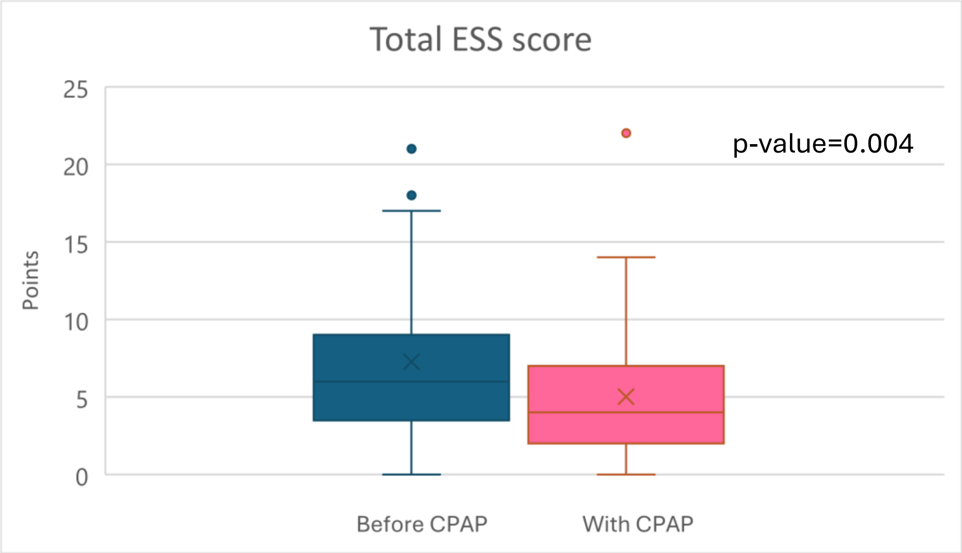
Total ESS score
Points
p-value=0.004
Before CPAP
With CPAP

Supplementary Figure 2. Four (A – daily functioning, B – social interactions, C – emotional functioning, and D – symptoms potentially due to obstructive sleep apnea) domains and the total score of the Calgary Sleep Apnea Quality of Life Index (SAQLI), and Epworth Sleepiness Scale (ESS) results before and after one month of continuous positive airway pressure (CPAP) therapy usage (n=53).
