# Supplementary material for: Adherence to CPAP Therapy in Obstructive Sleep Apnea: A Prospective Study on Quality of Life and Determinants of Use
Source: Eur J Investig Health Psychol Educ. 2024 Aug 27;14(9):2463–75. doi: 10.3390/ejihpe14090163 (PMC11431498; doi:10.3390/ejihpe14090163)
Supplement: Supplementary file 1 [file ejihpe-14-00163-s001.zip › Supplementary tables.pdf]

Table S1. Reliability analysis and the Cronbach's alpha for the daily functioning domain of Calgary Sleep Apnea Quality of Life Index

	**Scale mean if item deleted**	**Scale variance if item deleted**	**Corrected item-total correlation**	**Squared multiple correlation**	**Cronbach's alpha if item deleted**
Item A_I_1	30.0	160.9	0.65	0.52	0.91
Item A_I_2	30.9	160.2	0.75	0.80	0.91
Item A_I_3	31.1	164.2	0.69	0.78	0.91
Item A_I_4	30.4	165.5	0.67	0.62	0.91
Item A_II_1	29.7	159.2	0.75	0.67	0.91
Item A_II_2	30.3	164.6	0.69	0.64	0.91
Item A_II_3	30.3	165.0	0.70	0.77	0.91
Item A_II_4	30.3	165.0	0.64	0.62	0.91
Item A_III_1	30.5	166.5	0.57	0.73	0.92
Item A_III_2	30.5	163.3	0.66	0.80	0.91
Item A_III_3	30.2	160.0	0.75	0.65	0.91

**Cronbach's alpha**	**Cronbach's alpha based on standardized items**	**N of items**
0.92	0.9	11.0

Table S2. Reliability analysis and the Cronbach's alpha for the social interactions domain of Calgary Sleep Apnea Quality of Life Index

	Scale mean if item deleted	Scale variance if item deleted	Corrected item-total correlation	Squared multiple correlation	Cronbach's alpha if item deleted
Item B_1	32.8	205.4	0.64	0.55	0.90
Item B_2	33.9	211.7	0.57	0.61	0.90
Item B_3	33.8	203.2	0.74	0.67	0.90
Item B_4	33.3	202.6	0.68	0.70	0.90
Item B_5	33.3	201.3	0.63	0.54	0.90
Item B_6	33.2	200.9	0.67	0.56	0.90
Item B_7	33.2	208.1	0.61	0.63	0.90
Item B_8	32.8	202.1	0.66	0.70	0.90
Item B_9	33.8	214.4	0.61	0.58	0.90
Item B_10	33.9	212.4	0.70	0.59	0.90
Item B_11	34.0	213.4	0.60	0.46	0.90
Item B_12	33.6	215.4	0.49	0.60	0.91
Item B_13	33.9	214.2	0.58	0.53	0.90

Cronbach's alpha	Cronbach's alpha based on standardized items	N of items
0.91	0.9	13.0

Table S3. Reliability analysis and the Cronbach's alpha for the emotional functioning domain of Calgary Sleep Apnea Quality of Life Index

	Scale mean if item deleted	Scale variance if item deleted	Corrected item-total correlation	Squared multiple correlation	Cronbach's alpha if item deleted
Item C_1	27.3	151.7	0.76	0.67	0.94
Item C_2	27.7	156.0	0.70	0.59	0.94
Item C_3	27.7	153.9	0.84	0.77	0.93
Item C_4	27.1	151.2	0.84	0.83	0.93
Item C_5	27.1	153.1	0.73	0.64	0.94
Item C_6	27.6	155.8	0.75	0.65	0.94
Item C_7	26.9	147.6	0.80	0.84	0.93
Item C_8	27.2	148.4	0.84	0.83	0.93
Item C_9	27.7	151.9	0.81	0.78	0.93
Item C_10	27.7	161.7	0.53	0.53	0.94
Item C_11	27.8	161.8	0.61	0.65	0.94

Cronbach's alpha	Cronbach's alpha based on standardized items	N of items
0.94	0.9	11.0

Table S4. Reliability analysis and the Cronbach's alpha for the Cues to CPAP Use Questionnaire (CCUQ)

	**Scale mean if item deleted**	**Scale variance if item deleted**	**Corrected item-total correlation**	**Squared multiple correlation**	**Cronbach's alpha if item deleted**
Item 1 cues	13.2	20.7	0.30	0.60	0.59
Item 2 cues	14.1	17.9	0.45	0.43	0.55
Item 3 cues	14.2	23.3	-0.05	0.17	0.69
Item 4 cues	13.4	19.5	0.40	0.59	0.57
Item 5 cues	15.2	20.5	0.29	0.24	0.60
Item 6 cues	13.8	20.2	0.23	0.29	0.61
Item 7 cues	13.7	19.0	0.44	0.33	0.56
Item 8 cues	13.8	19.7	0.39	0.28	0.57
Item 9 cues	15.3	19.5	0.39	0.35	0.57

**Cronbach's alpha**	**Cronbach's alpha based on standardized items**	**N of items**
0.62	0.6	9.0
